# Supplementary material for: Factor H binding proteins protect division septa on encapsulated Streptococcus pneumoniae against complement C3b deposition and amplification
Source: Nat Commun. 2018 Aug 23;9:3398. doi: 10.1038/s41467-018-05494-w (PMC6107515; doi:10.1038/s41467-018-05494-w)
Supplement: Supplementary file 1 — Supplementary Information [file 41467_2018_5494_MOESM1_ESM.pdf]

Supplementary Information

Factor H binding proteins protect division septa on encapsulated *Streptococcus pneumoniae* against complement C3b deposition/amplification

Pathak et al.

Supplementary Fig. 1.

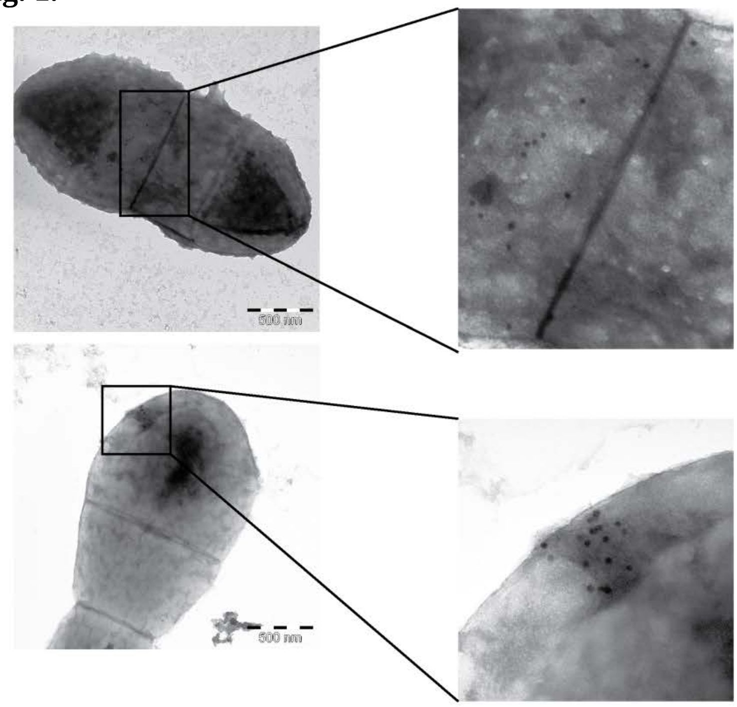

BHN418

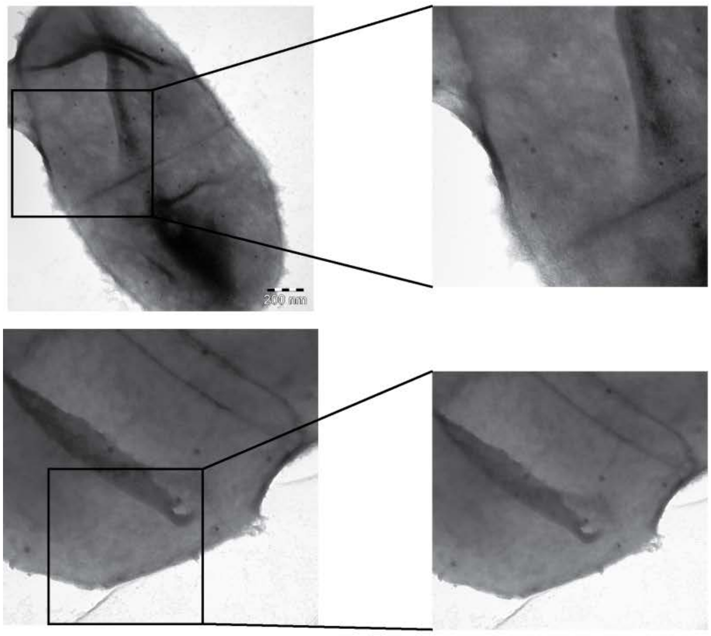

BHN418Δ*capsule*

TEM images of immunogold staining of C3b on bacteria. Representative TEM images of immunogold staining of C3b on wt BHN418 and non-encapsulated BHN418 (BHN418Δ*capsule*) after incubation with 20% normal human serum. Zoomed parts of the images are shown for better visibility of gold particles.

Supplementary Fig. 2.

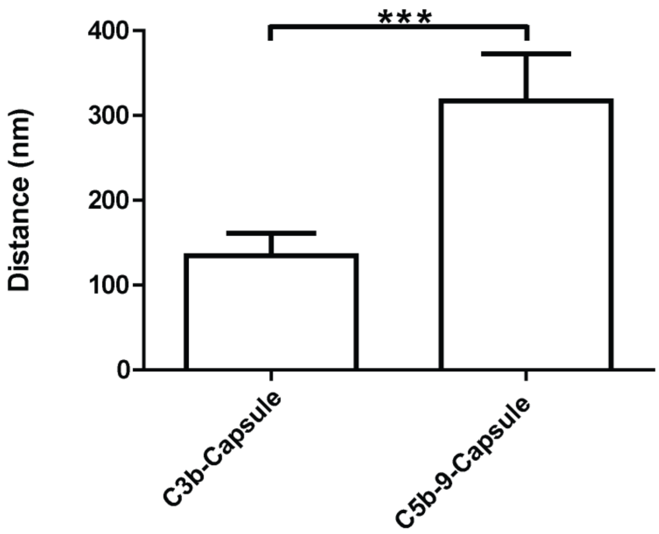

Calculations of the distance between the capsule, and C3b or C5b-9, respectively. The distances between the capsule and C3b (shown in figure 2c) or C5b-9 (shown in figure 2d) were calculated by manually drawing a line, in the image, perpendicular to the capsule and the complement to create a line profile. The distance was then estimated as the distance between the peaks in the line profile of the capsule and complement respectively. The distance between the capsule and C5b-9 (n=37) showed a larger distance in comparison to the capsule and C3b (n=53). Graph shows Mean ± SD (two way student t-test) ***, $p < 0.001$.

Supplementary Fig. 3.

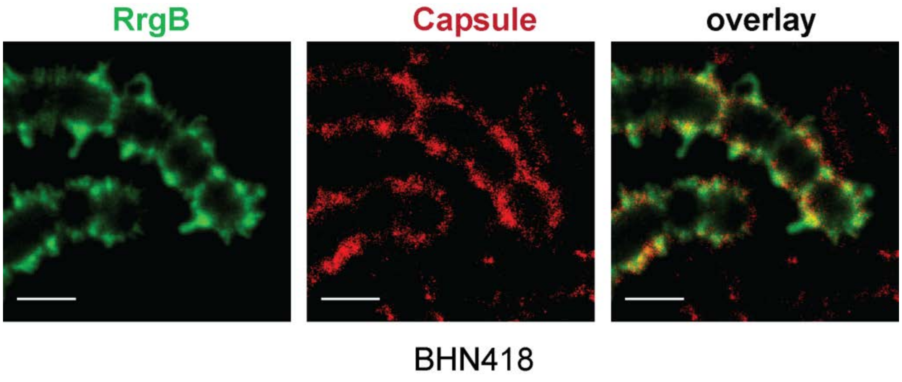

Localization of RrgB on the pneumococcal strain BHN418.

Super-resolution STED microscopy images of RrgB on pneumococcal strain BHN418. Bacteria were sequentially stained with goat anti-RrgB serum followed by Atto647N labeled secondary antibody (green). Capsule was detected using rabbit anti-6B capsule serum and anti-rabbit Alexa fluor 594 antibody (red). Scale bar = 1µm

Supplementary Fig. 4.

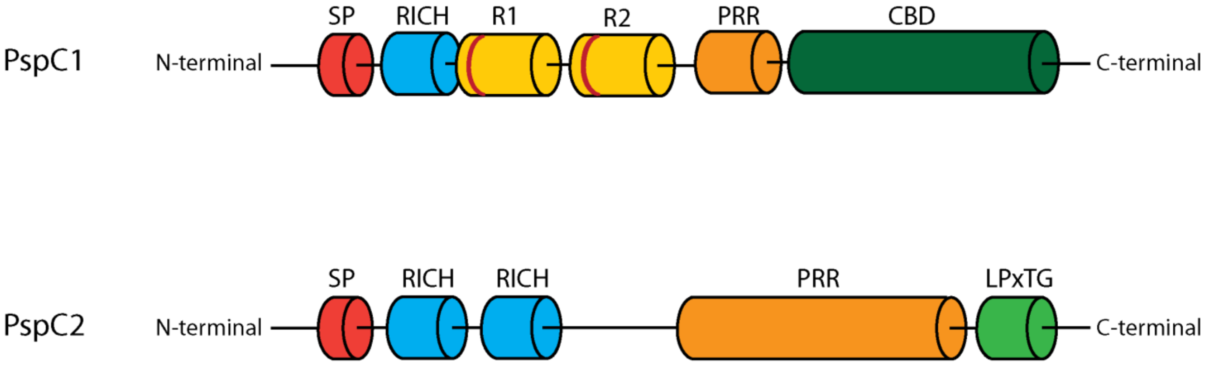

Model structure of PspC1 and PspC2 from BHN418. PspC2 contains two RICH domains (Pfam-PF05062) in its N-terminal alpha helical part in comparison to one RICH domain in PspC1. R1 and R2 domains, containing conserved pIgR binding motif (represented by red lines), are present in PspC1, but absent in PspC2.

Abbreviations: PRR-proline rich repeats, SP-signal peptide, CBD-choline binding domain, LPxTG-LPxTG anchor domain

Supplementary Fig. 5.

PspC1 BHN418

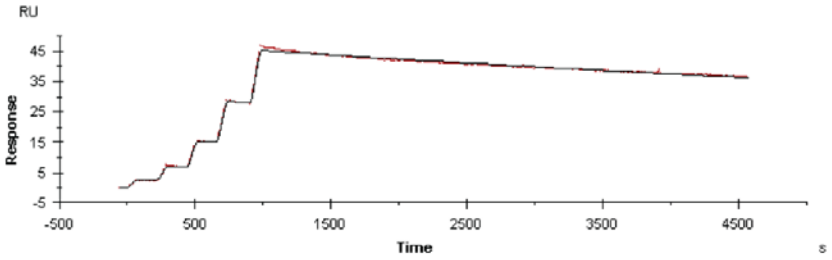

Curve	ka (1/Ms)	kd (1/s)	KD (M)	Rmax (RU)	Conc (M)	tc	Flow (ul/min)	kt (RU/Ms)	RI (RU)	Chi² (RU²)	U-value
Cycle: 6	6,854E+5	6,221E-5	9,076E-11	65,55	9,375E-10	1,726E+9	30,00	5,363E+9	-0,03424	0,126	1
					1,875E-9				-0,3875		
					3,750E-9				-0,4645		
					7,500E-9				-0,7131		
					1,500E-8				-0,6823		

PspC2 BHN418

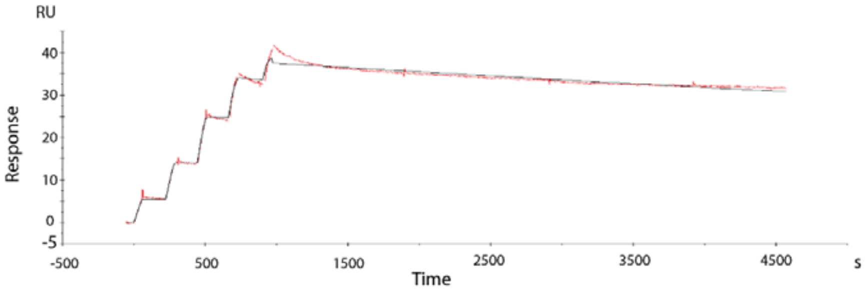

Curve	ka (1/Ms)	kd (1/s)	KD (M)	Rmax (RU)	Conc (M)	tc	Flow (ul/min)	kt (RU/Ms)	RI (RU)	Chi² (RU²)	U-value
Cycle: 4	4,11E+06	5,47E -05	1,33E -11	37,91	6,25E -10	6,30E+13	30	1,96E+14	0,2367	0,455	2
					1,25E -09				-0,1538		
					2,50E -09				-0,5736		
					5,00E -09				-0,2998		
					1,00E -08				1,387		

PspC D39

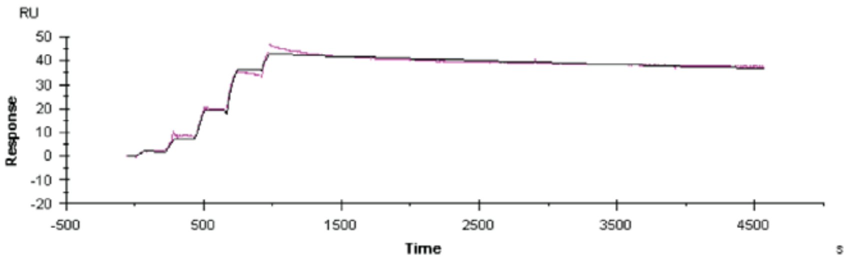

Curve	ka (1/Ms)	kd (1/s)	KD (M)	Rmax (RU)	Conc (M)	tc	Flow (ul/min)	kt (RU/Ms)	RI (RU)	Chi² (RU²)	U-value
Cycle: 6	4,836E+5	4,281E-5	8,854E-11	43,03	1,600E-9	1,752E+21	30,00	5,443E+21	-0,1647	0,754	2
					4,810E-9				-0,2538		
					1,440E-8				0,02706		
					4,330E-8				-2,374		
					1,300E-7				-1,903		

Surface plasmon resonance/BIACORE analysis of PspC-FH interactions.
Representative sensorgrams of surface plasmon resonance analysis of PspC-FH interactions for three different PspC alleles. Response (resonance unit-RU) versus time plot was obtained by injecting five increasing concentrations of recombinant PspC proteins. Colored curves were obtained by fitting data with a 1:1 binding model. See Methods for a more detailed description.

Supplementary Fig. 6.

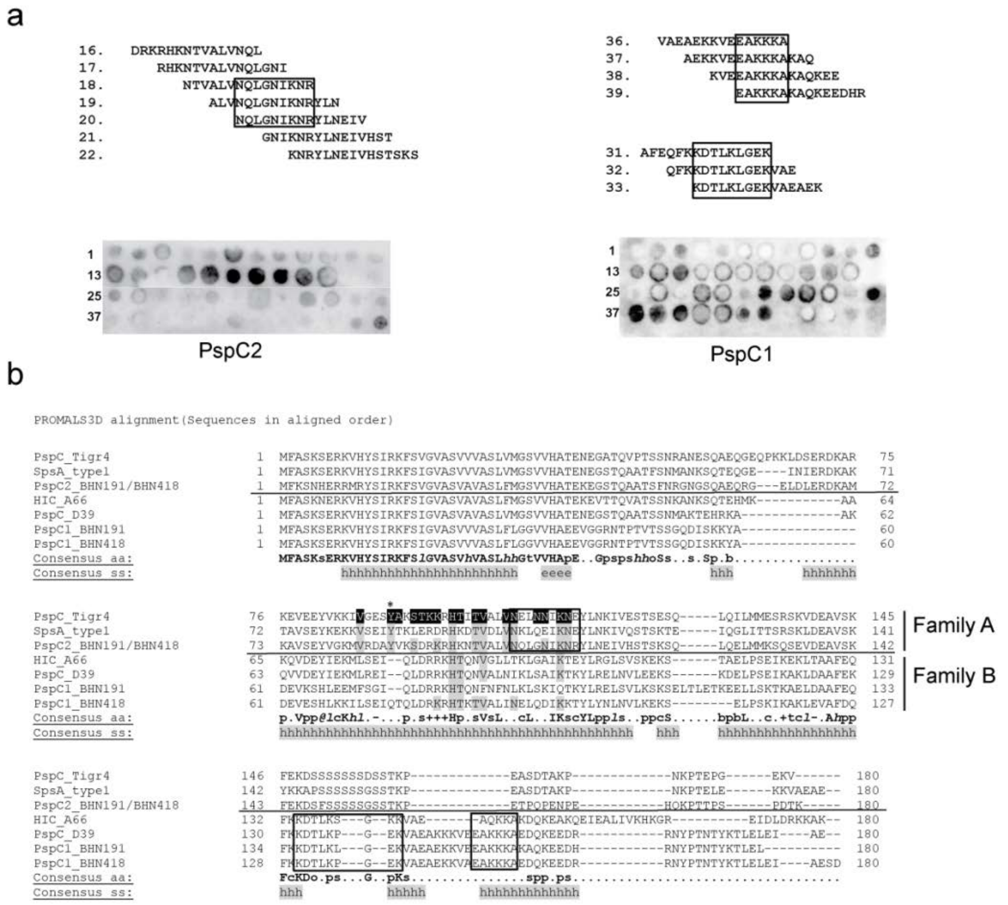

Identification of the FH binding domain of PspC1 and PspC2.

(a) An array of 48 overlapping peptides was synthesized and covalently spotted onto a cellulose membrane covering the N terminal domain of PspC1 and PspC2. Each spot contained a 15 amino acids long peptide with three overlapping amino acids to the next spot. Spots showing more than 80 percent binding were considered as full binders. Sequences of amino acids from the binding peptides are shown with binding motif represented by an empty box.

(b) Structure based alignment of N terminal domains of different PspCs. The N terminal sequence of two copies of PspC present in clinical isolates showing a high FH binding level (PspC1 and PspC2) and other members of the PspC family (PspC from TIGR4, SpsA_type1[S2], D39 and HIC[S3] were aligned with the structure based alignment tool PROMALS3D[S4]. The recently described three dimensional structure of the FH binding part of TIGR4 PspC was used as a template for the structure based alignment. All 17 residues of PspC in TIGR4 shown to interact with human FH are shaded in black. Shaded in grey are the residues that are conserved in other PspCs. Key residue Tyrosine[90] described in the crystal structure is indicated by a star. Family A and family B of PspCs are labeled accordingly with a line separating both families. Empty boxes show conserved residues of FH binding motifs in PspC1 and PspC2 found by peptide array analysis. Consensus amino acids and consensus secondary structures are mentioned in the last rows.

Supplementary Fig. 7.

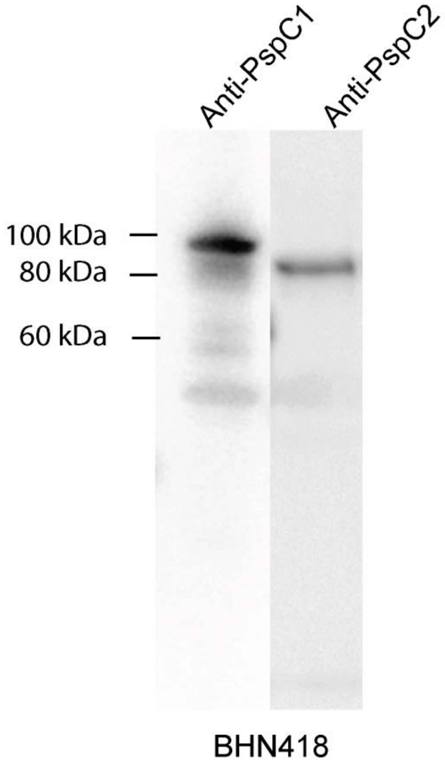

Specificity of anti-PspC1 and anti-PspC2 antibodies.
Western blot analysis showing the specificity of the anti-PspC1 and anti-PspC2 antibodies used in the study when probed against cell lysate of wt BHN418. Calculated molecular weight of PspC1 and PspC2 is 80.93 kDa and 66.92 kDa, respectively. Full blots are shown in Supplementary Fig. 24a.

Supplementary Fig. 8.

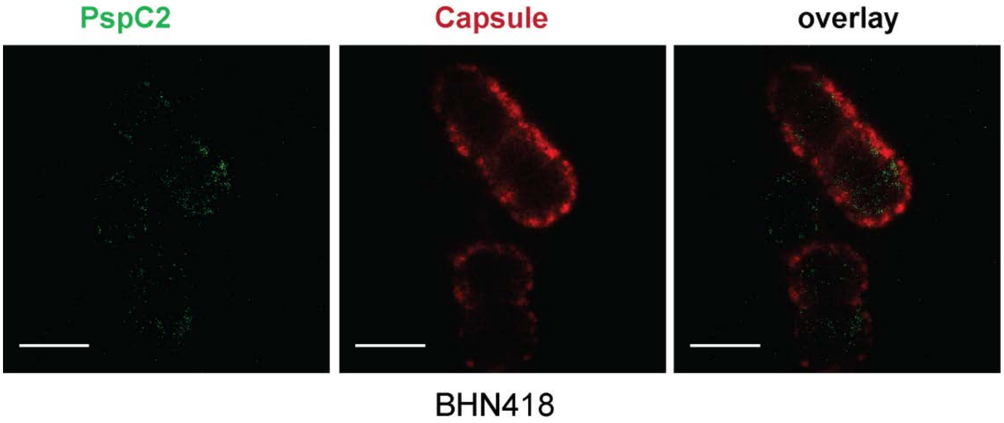

Localization of PspC2 in relation to the capsule on pneumococcal strain BHN418.
Super-resolution STED microscopy images of PspC2 and the capsule on strain BHN418. Bacteria were sequentially stained with rabbit anti-PspC2 serum followed by Atto647N labeled secondary antibody (green). The capsule was detected using rabbit anti-6B capsule serum and anti-rabbit Alexa fluor 594 antibody (red). Scale bar = 1µm

Supplementary Fig. 9.

a

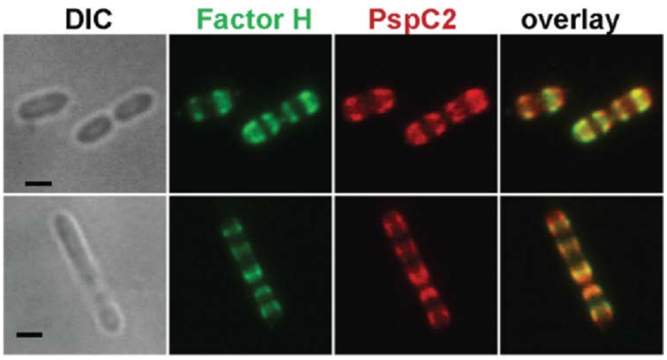

BHN191 (choline chloride treated)

b

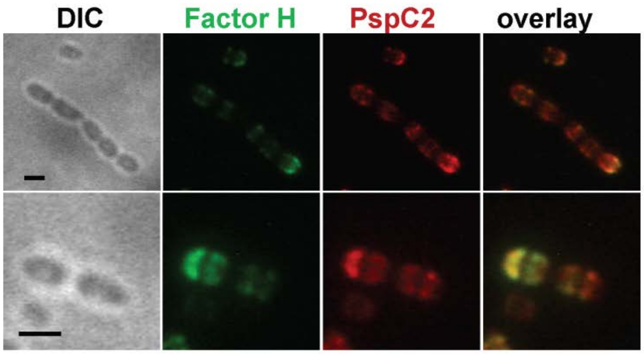

BHN418Δ*pspC1* (choline chloride treated)

Localization of FH and PspC2 after choline chloride treatment of the pneumococcal strain BHN191, and of *pspC* mutants in BHN418.

Representative immunofluorescence images of PspC2 localization in choline chloride treated strains: **(a)** BHN191, belonging to the same serotype (6B) and sequence type (CC138) as BHN418, and of **(b)** the isogenic mutant in *pspC1* in BHN418, BHN418*ΔpspC1*. Bacteria were incubated with purified human FH. FH and PspC2 were stained as in Fig. 4c. There was no detectable FH staining when BHN418*ΔpspC2* or BHN418*ΔpspC1ΔpspC2* were treated with choline chloride. **a-b** Scale bar = 1µm

Supplementary Fig. 10.

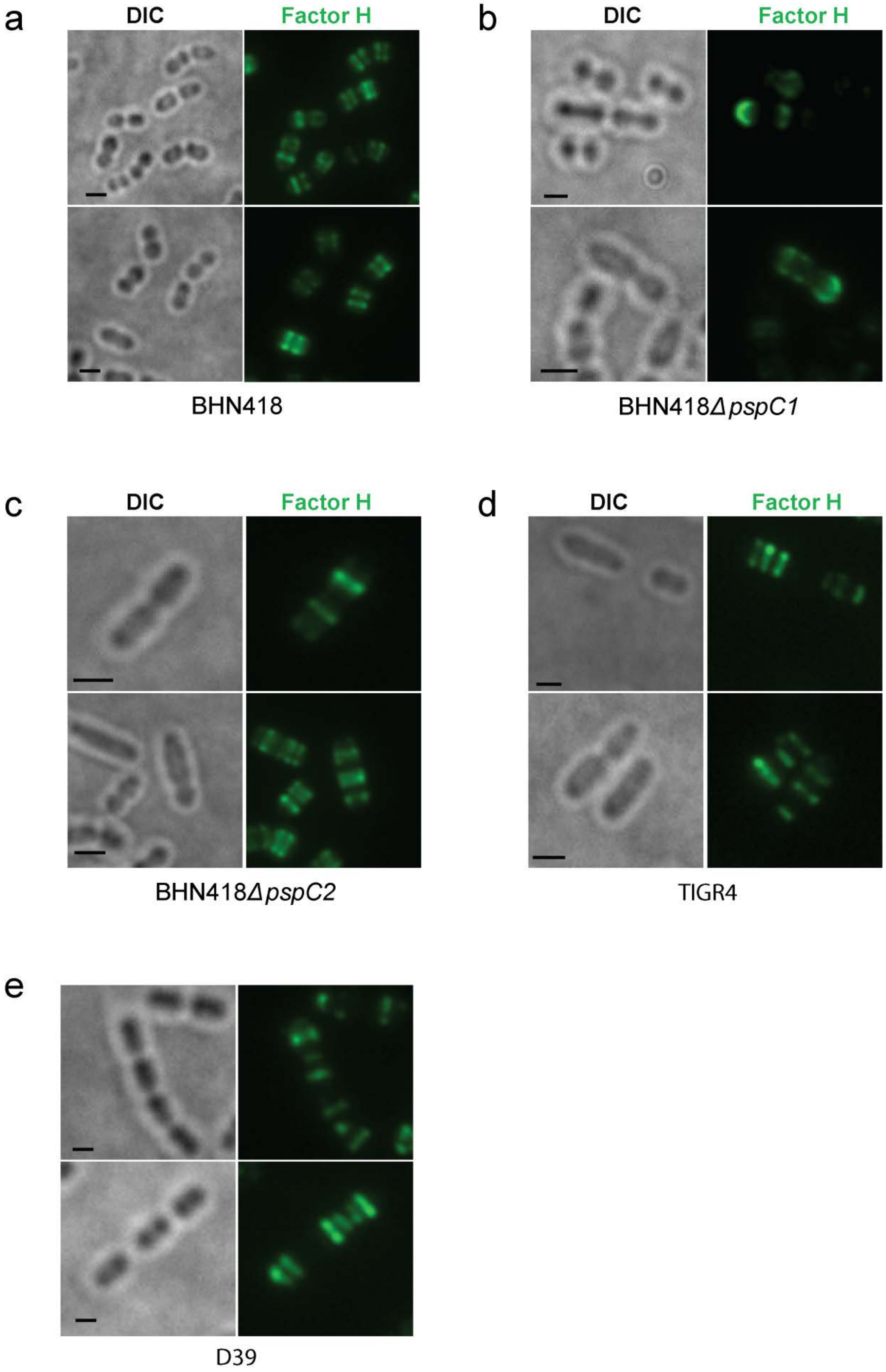

Localization of factor H (FH) on different pneumococcal strains incubated with 20% NHS.

Representative immunofluorescence images of FH deposition on strains: **(a)** BHN418 and its isogenic **(b)** *pspC1* and **(c)** *pspC2* mutants, as well as on strains **(d)** TIGR4 and **(e)** D39. The strains were incubated with 20% NHS, and FH was detected using a polyclonal goat anti-FH antibody and a FITC-labeled rabbit anti-goat IgG secondary antibody (green). Scale bar = 1µm

Supplementary Fig. 11.

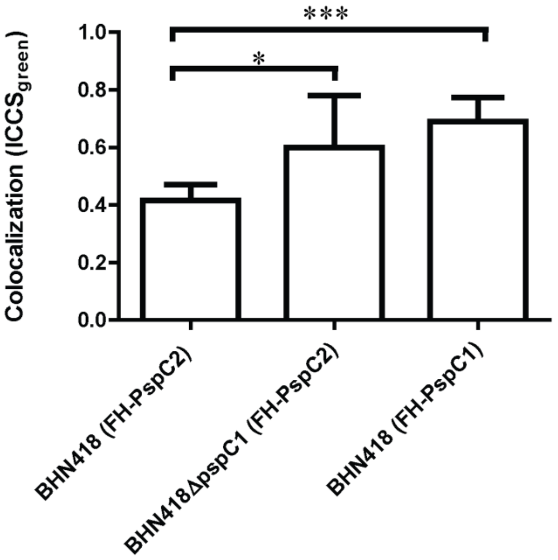

Calculation of co-localization between PspC1 and PspC2 and FH.

The co-localization coefficient for the staining of PspC1 and PspC2 and FH, as shown in Figure 4, was calculated (see Methods for a more detailed description). PspC2 and FH showed a low co-localization coefficient in wt BHN418, while a significantly higher co-localization coefficient was found in the mutant lacking PspC1, BHN418Δ*pspC1*. FH and PspC1 in wt BHN418 showed a high co-localization coefficient. Number of cells included in the analyses: PspC2: BHN418 (n=106), and BHN418Δ*pspC1* (n=95). PspC1: BHN418 (n=111). Graph shows Mean ± SD (student t-test) *, $p < 0.05$ ***, $p < 0.001$.

Supplementary Fig. 12.

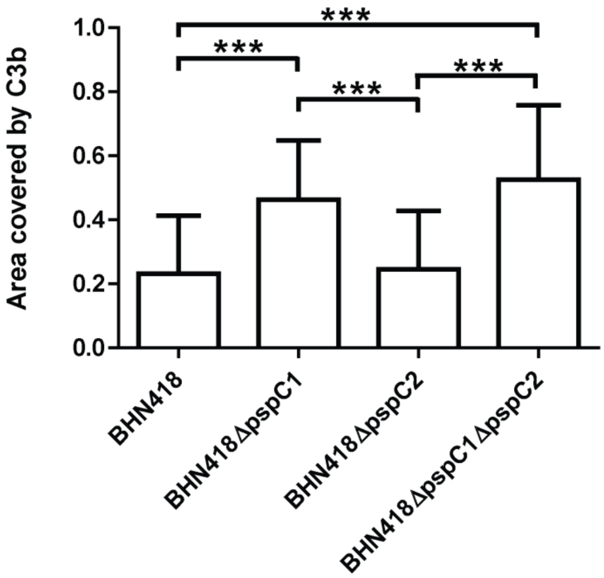

Calculations of the bacterial surface covered by C3b. The area covered by C3b was calculated (see Methods for a more detailed description) for the images shown in Figure 5a. Wild type BHN418 (n=34) and BHN418*ΔpspC2* (n=33) showed significantly lower areas covered by C3b in comparison to the mutants BHN418*ΔpspC1* (n=48) and BHN418 *ΔpspC1ΔpspC2* (n=32). Graph shows Mean ± SD (student t-test) ***, $p < 0.001$

Supplementary Fig. 13.

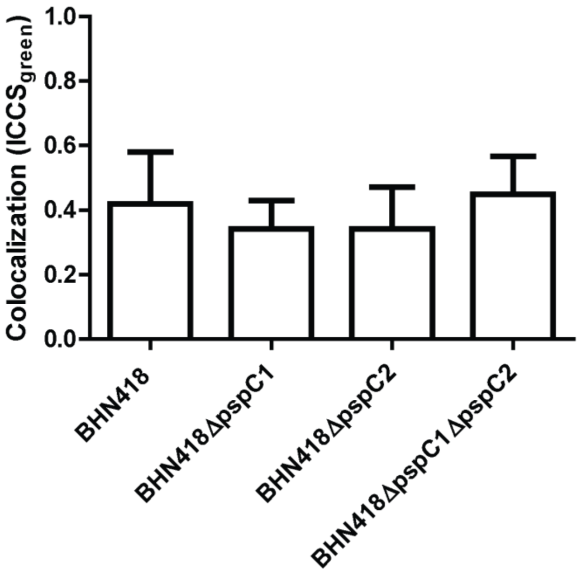

Calculation of co-localization between the capsule and C3b.
The co-localization coefficient was calculated (see Methods for a more detailed description) for the capsule and C3b for images shown in Figure 5. The capsule and C3b showed a low co-localization coefficient for wt BHN418 and its isogenic *pspC* mutants. Number of cells included in the analyses: BHN418 (n=32), BHN418Δ*pspC1* (n=57), BHN418*ΔpspC2* (n=28), BHN418*ΔpspC1ΔpspC2* (n=35). No significant differences in co-localization were found according to the student t-test. Graph shows Mean ± SD.

Supplementary Fig. 14.

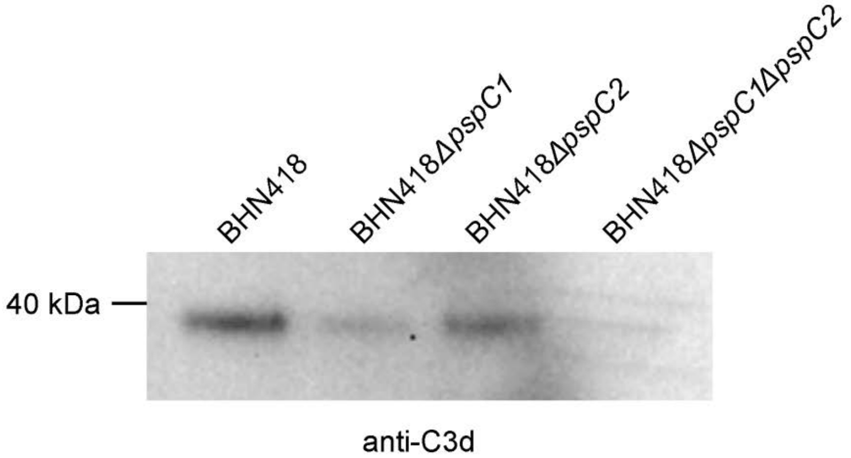

Detection of the degradation product C3d.
Western blot analysis showing deposited C3d in wt BHN418 and in its isogenic *pspC* mutants, BHN418*ΔpspC1,* BHN418*ΔpspC2* and BHN418*ΔpspC1ΔpspC2*. Full blot is shown in Supplementary Fig. 24b.

Supplementary Fig. 15.

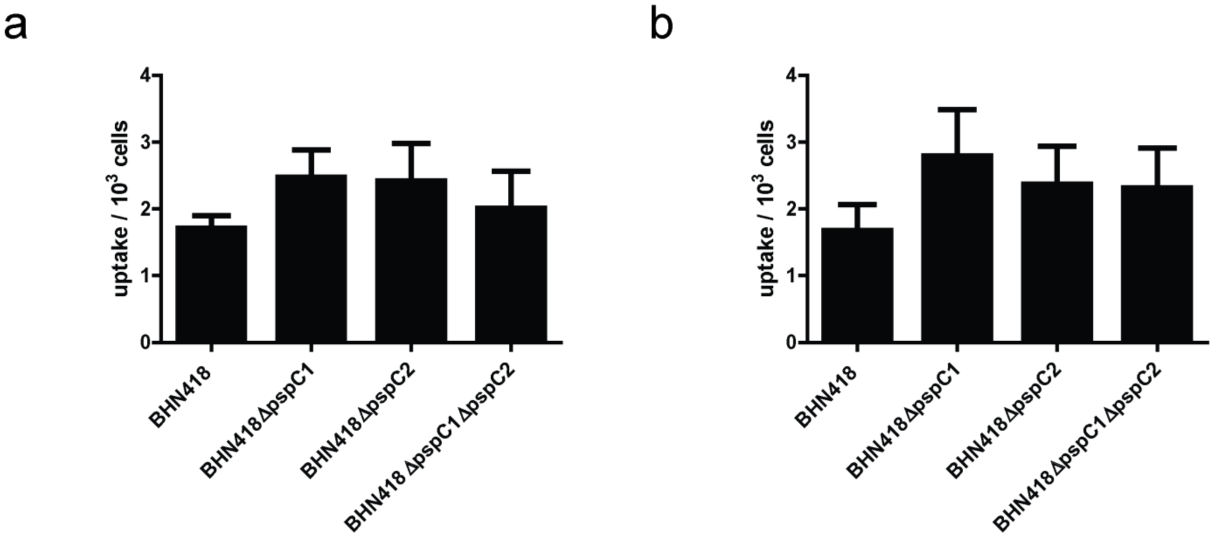

Pneumococcal uptake by THP-1 cells in the presence of pure FH and pure C3b.
Uptake by THP-1 derived macrophages of pneumococcal strain BHN418 or its isogenic *pspC* deletion mutants in the presence of **(a)** purified FH or **(b)** purified C3b. No significant difference was found between the strains. Graph shows Mean ± SEM.

Supplementary Fig. 16.

```
CLUSTAL O(1.2.4) multiple sequence alignment


PspC6.13        MFASKNERKVHYSIRKFSIGVASVAVASLFMGSVVHA
PspC8.1         MFASKSERKVHYSIRKFSIGVASVAVASLVMGSVVHA
PspC8.2         MFASKSERKVHYSIRKFSIGVASVAVASLVMGSVVHA
PspC8.3         MFASKSERKVHYSIRKFSIGVASVAVASLVMGSVVHA
PspC8.4         MFASKSERKVHYSIRKFSIGVASVAVASLVMGSVVHA
PspC11.2        MFASKNERKVHYSIRKFSIGVASVAVASLFMGSVVHA
PspC11.3        MFASKNERKVHYSIRKFSIGVASVAVASLFMGSVVHA
PspC2.5         MFASKSERKVHYSIRKFSIGVASVAVASLVMGSVVHA
PspC3.1         MFASKSERKVHYSIRKFSIGVASVAVASLVMGSVVHA
PspC3.6         MFASKSERKVHYSIRKFSIGVASVAVASLVMGSVVHA
PspC3.7         MFASKNERKVHYSIRKFSIGVASVAVASLFMGSVVHA
PspC3.8         MFASKSERKVHYSIRKFSIGVASVAVASLVMGSVVHA
PspC3.9         MFASKNERKVHYSIRKFSIGVASVAVASLFMGSVVHA
PspC3.10        MFASKNERKVHYSIRKFSIGVASVAVASLFMGSVVHA
PspC3.11        MFASKSERKVHYSIRKFSIGVASVAVASLVMGSVVHA
PspC3.12        MFASKNERKVHYSIRKFSIGVASVAVASLFMGSVVHA
PspC6.14        MFASKSERKVHYSIRKFSVGVASVVVASLFLGRVVHA
PspC6.4         MFASKSERKVHYSIRKFSVGVASVVVASLFLGRVVHA
PspC5.2         MFASKSERKVHYSIRKFSIGVASVVVASLVMGSVVHA
PspC5.1         MFASKSERKVHYSIRKFSIGVASVVVASLVMGSVVHA
PspC3.13        MFASKSERKVHYSIRKFSIGVASVVVASLVMGSVVHA
PspC3.5         MFASKKERKVHYSIRKFSIGVASVVVASLFMGSVVHA
PspC2.4         MFASKSERKVHYSIRKFSIGVASVVVASLVMGSVVHA
PspC2.3         MFASKSERKVHYSIRKFSIGVASVVVASLVMGSVVHA
PspC2.2         MFASKSERKVHYSIRKFSVGVASVVVASLVMGSVVHA
PspC1.1         MFASKSERKVHYSIRKFSIGVASVAVASLFLGGVVHA
PspC6.11        MFASKSERKVHYSIRKFSIGVASVAVASLFLGGVVHA
PspC11.1        MFKSNHERRMRYSIRKFSVGVASVAVASLFMGSVVHA
PspC9.4         MFKSNHERRMRYSIRKFSVGVASVAVASLFMGSVVHA
PspC9.3         MFKSNHERRMRYSIRKFSVGVASVAVASLFMGSVVHA
PspC9.2         MFKSNHERRMRYSIRKFSVGVASVAVASLFMGSVVHA
PspC9.1         MFKSNHERRMRYSIRKFSVGVASVAVASLFMGSVVHA
PspC10.1        MFKSNHERRMRYSIRKFSVGVASVAVASLFMGSVVHA
PspC6.8         MFASKNERKVHYSIRKFSIGVASVAVASLFLGGVAHA
PspC6.1         MFASKSERKVHYSIRKFSIGVASVAVASLFLGGVAHA
PspC6.12        MFASKSERKVHYSIRKFSIGVASVVVASLFLGGVVHA
PspC6.10        MFASKSERKVHYSIRKFSVGVASVVVASLFLGGVVHA
PspC6.7         MFASKSERKVHYSIRKFSIGVASVVVASLFLGGVVHA
PspC6.6         MFASKSERKVHYSIRKFSVGVASVVVASLFLGGVVHA
PspC6.2         MFASKSERKVHYSIRKFSVGVASVVVASLFLGGVVHA
PspC6.9         MFASKSERKVHYSIRKFSIGVASVVVASLFLGGVVHA
PspC6.5         MFASKSERKVHYSIRKFSIGVASVVVASLFLGGVVHA
PspC4.1         MFASKSERKVHYSIRKFSIGVASVVVASLFLGGVVHA
PspC7.1         MFKSNYERKMCYSIRKFSIGVASVAVASLVMGSVVHA
PspC7.2         MFKSNYERKMCYSIRKFSIGVASVAVASLVMGSVVHA
PspC7.3         MFKSNYERKMCYSIRKFSIGVASVAVASLVMGSVVHA
PspC7.4         MFKSNYERKMCYSIRKFSIGVASVAVASLVMGSVVHA
                ** *  **   ******* ***** ****  * * **
```

Sequence alignment of signal peptides from different alleles of PspC.
Different signal peptide sequences were aligned using Clustal omega. Conserved residues are shown in green. The box shows the twin arginine motif present specifically in PspC2 or similar alleles.

Supplementary Fig. 17.

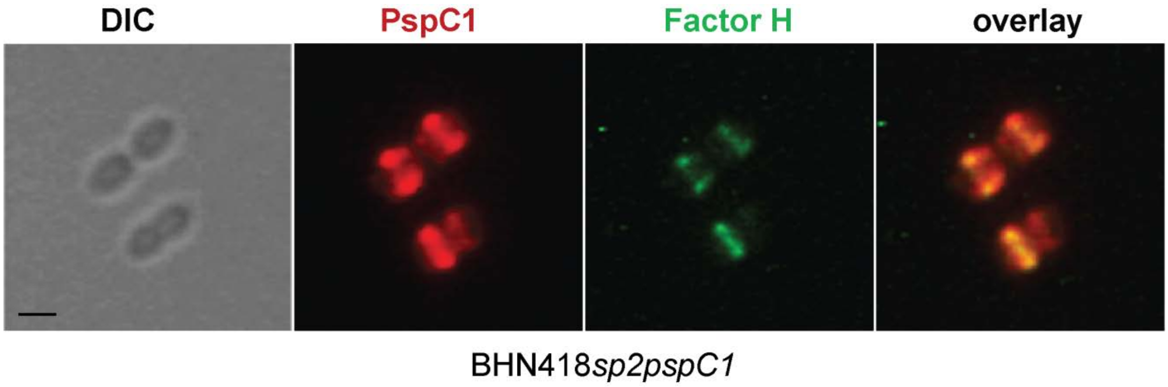

PspC localization and FH binding in the signal peptide switch mutant BHN418*sp2pspC1*. Representative immunofluorescence images of PspC1 and FH staining of the SP switch mutant BHN*sp2pspC1*. FH (green) was detected using a polyclonal goat anti-FH antibody and a FITC-labeled rabbit anti-goat IgG secondary antibody while PspC1 (red) was stained with anti PspC1 antiserum and anti-mouse Alexa fluor 594 secondary antibody. Scale bar = 1µm

Supplementary Fig. 18.

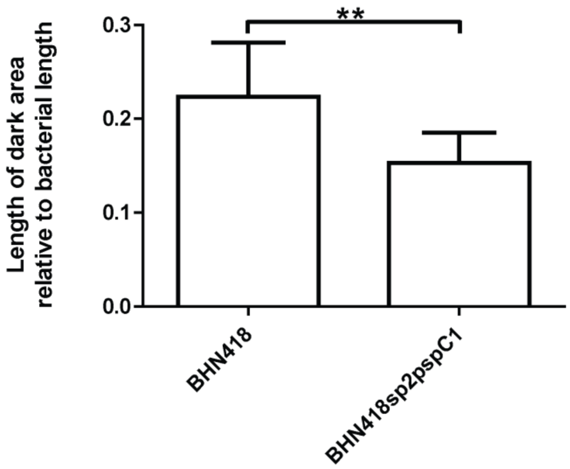

Calculation of the area not covered by the PspC1 signal in the color map shown in Figure 7c. The distance between the PspC1 bands as the full width at half minimum with respect to the lowest peak was calculated and was divided by the total length of the bacterium for BHN418 and the signal peptide mutant BHN418sp2*pspC1*. The length of the darker area as shown in the color map in Figure 7c was larger in wt BHN418 in comparison to BHN418sp2*pspC1*. Graph shows Mean ± SD (student t-test) **, $p < 0.01$.

Supplementary Fig. 19.

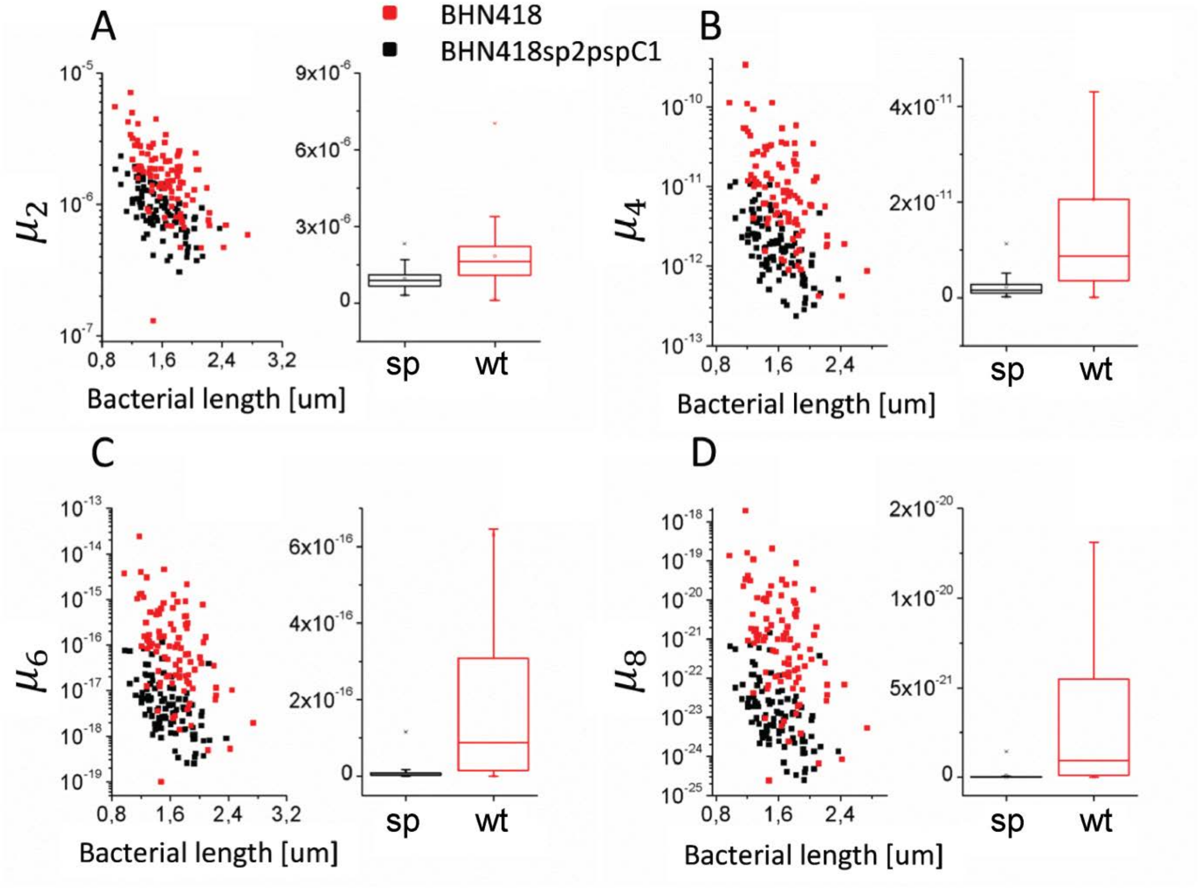

Higher order moments for analyzing "peak sharpness" of fluorescence trace in STED images.

Calculated higher order moments, μ_m, from the projected fluorescence profiles of wt BHN418 (red) and mutant BHN418sp2*pspC1*(black) bacteria (Figure 7C) are shown. The μ_m values are shown as a **s**catterplot with a corresponding boxplot of the higher order moments 2, 4, 6 and 8. Each point in the scatterplot corresponds to the calculated μ_m versus the length of each bacteria, and the corresponding boxplot shows the average of μ_m for all bacteria at the given order. Error bars represent 95% standard deviation. **(a)** Moments at order 2 (corresponding to the variance of the fluorescence trace along the bacteria). **(b)** Moments at order 4 (sometimes referred to askurtosis). **(c)** Moments at order 6. **(d)** Moments at order 8. The relative spread in moments for wild type BHN418 (wt) increases with increasing order *m*, while for the signal peptide mutant BHN418sp2*pspC1* (SP) the average moment goes to zero as the order increases. This suggests that the fluorescence intensity traces along wt bacteria deviate more from the mean than those for the SP mutants, indicating that the fluorescence peaks along wt bacteria are narrower/sharper than for the SP mutant. This in turn indicates that PspC1 form more of a ring structure in wt bacteria, and is more spread out along the bacteria in the SP mutant.

Supplementary Fig. 20.

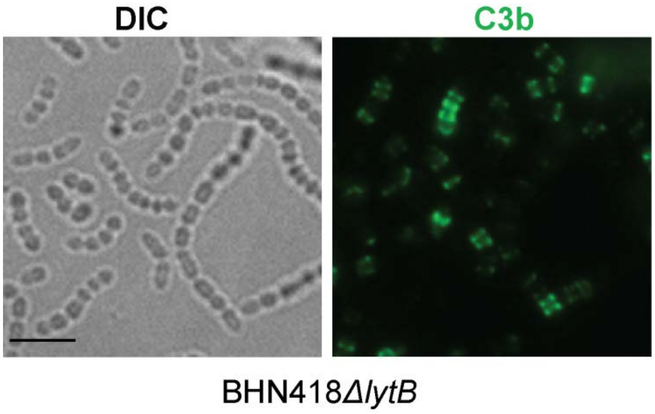

Complement deposition pattern on the chain forming LytB mutant of BHN418 (BHN418*ΔlytB*).

Representative immunofluorescence images of C3b deposition on chains of BHN418*ΔlytB*. C3b was stained using goat anti-C3 antibody followed by incubation with anti-goat Alexa fluor 488 secondary antibody (green). Scale bar = 5µm

Supplementary Fig. 21.

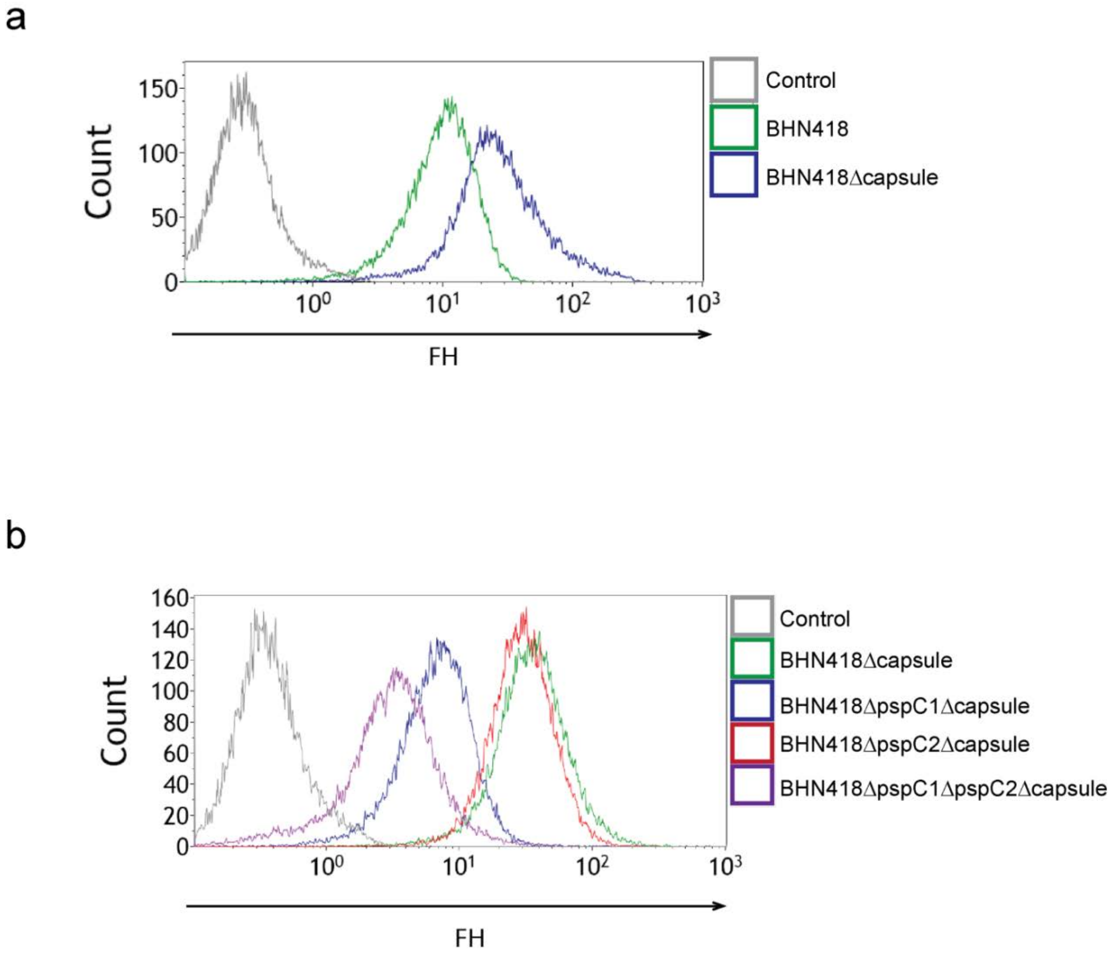

The pneumococcal capsule affects FH binding.

Representative histogram of FH binding to **(a)** wt BHN418 and its isogenic non-encapsulated mutant as well as **(b)** its isogenic *pspC* deletion mutants using flow cytometry. Bacteria were incubated with purified human FH and stained as in Fig. 4a. Bacteria incubated without FH were used as a control for each strain. The histogram shown is representative of three independent experiments.

Supplementary Fig. 22.

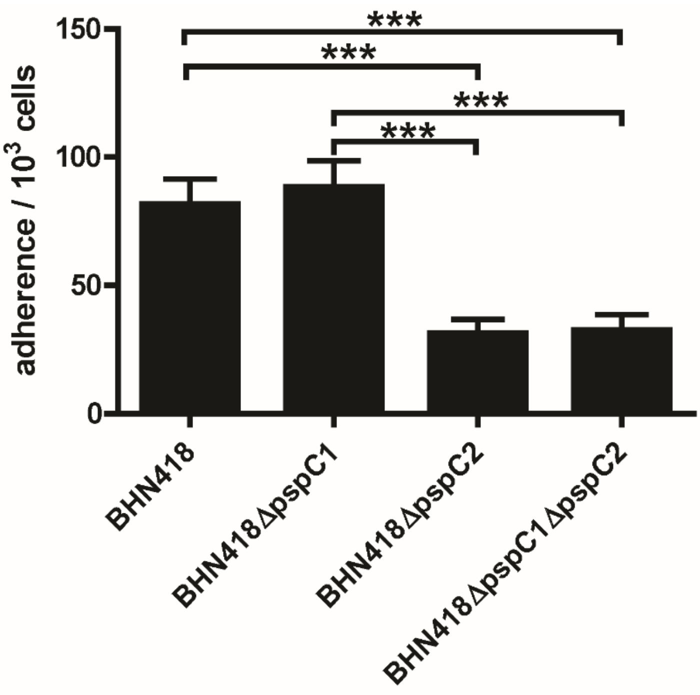

Pneumococcal adhesion to Detroit nasopharyngeal epithelial cells.
Adherence to Detroit cells of wild type BHN418 or its isogenic *pspC* mutants is shown. Graph shows mean ± SEM of four independent experiments. ***, $p < 0.001$.

Supplementary Fig. 23.

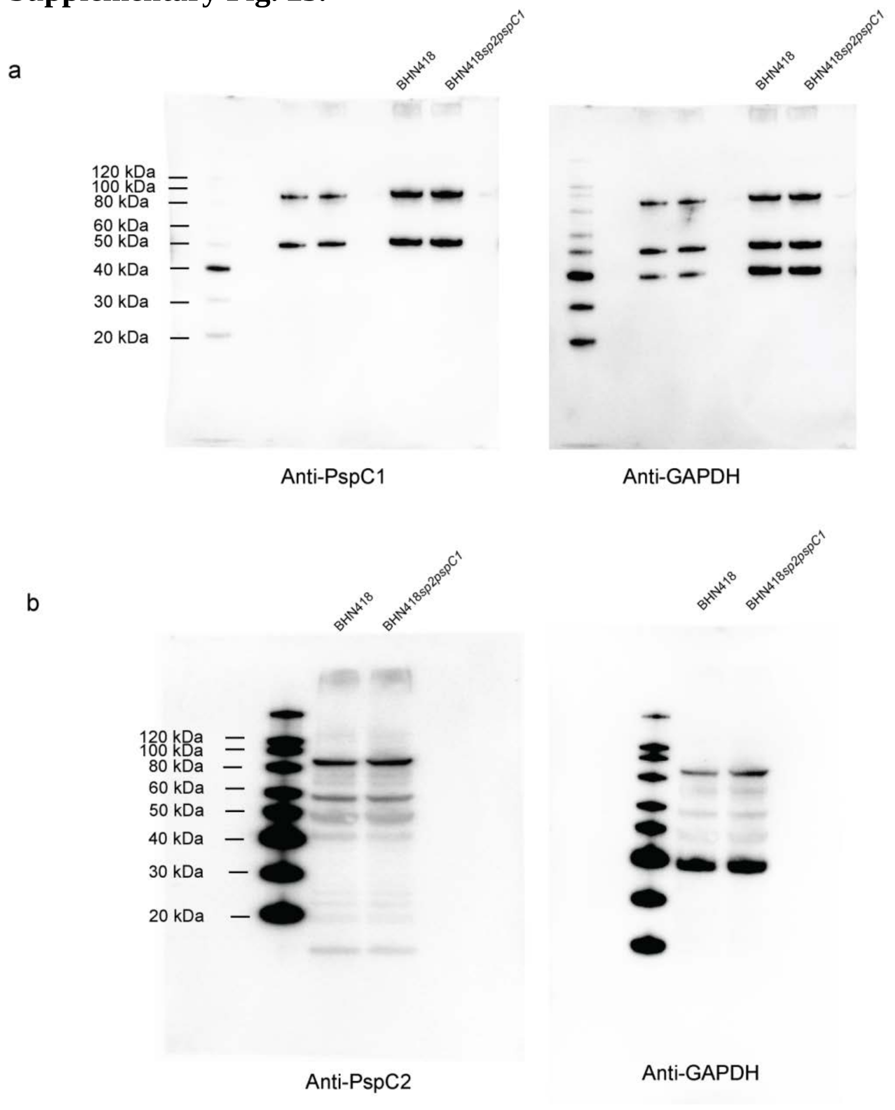

Full blots of western blot analysis showing the expression level of PspC1 and PspC2 in figure 7d (**a**) PspC1 in wt BHN418 and in the signal peptide switch mutant, BHN418*sp2pspC1* (left). An additional lower band was detected in a few blots when stained with anti-PspC1 antibody is believed to be proteolytic cleavage product of PspC1 where choline binding domain is cleaved. GAPDH was used as a loading control (right). (**b**) PspC2 in wt BHN418 and in the signal peptide switch mutant, BHN418*sp2pspC1* (left). GAPDH was used as a loading control (right). An additional lower band was detected in few blots when stained with anti-PspC1 antibody as detected is believed to be proteolytic cleavage product of PspC1 where choline binding domain is cleaved.

Supplementary Fig. 24

a

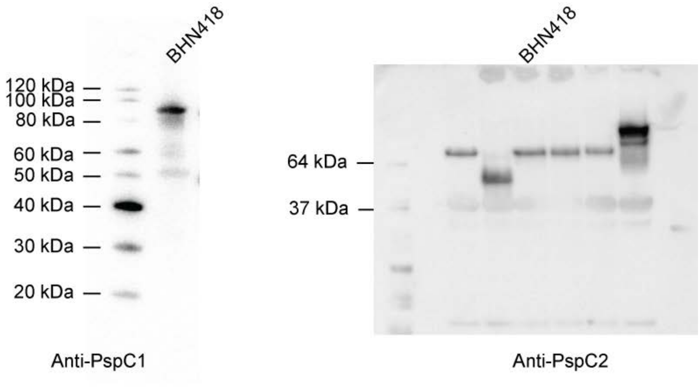

b

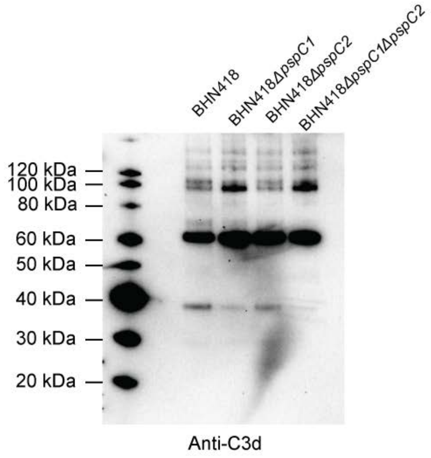

Full blots of
(a) specificity of anti-PspC1 and anti-PspC2 antibodies in Suppl. Figure 7.
Western blot analysis showing the specificity of the anti-PspC1 (left) and anti-PspC2 (right) antibodies used when probed against cell lysate of wt BHN418. Calculated molecular weight of PspC1 and PspC2 is 80.93 kDa and 66.92 kDa, respectively.
(b) detection of the degradation product C3d in Suppl. Figure 14.
Western blot analysis showing deposited C3d in wt BHN418 and in its isogenic *pspC* mutants, BHN418*ΔpspC1,* BHN418*ΔpspC2* and BHN418*ΔpspC1ΔpspC2*.

Supplementary Tables

Supplementary Table 1. Strains used in this study.

Strain	Characteristics	Ref./source
WT-TIGR4	serotype 4	www.tigr.org
TIGR4Δ*pspC*	TIGR4 PspC::erm Ery^R	This study
BNH657	D39, serotype 2	This study
BHN191	clinical isolate, serotype 6B	S1[a]
BHN418	clinical isolate, serotype 6B	S1[a]
BHN418Δ*pspC1*	BHN418 *pspC1::erm* Ery^R	This study
BHN418Δ*pspC2*	BHN418 *pspC2::erm* Ery^R	This study
BHN418Δ*pspC1*Δ*pspC2*	BHN418 *pspC1::erm* Ery^R *pspC2::kan* Kan^R	This study
BHN418*sp2pspC1*	BHN418 *pspC1*signal petide switch*::kan* Kan^R	This Study
BHN418Δ*capsule*	BHN418 *Cps::tet* Tet^R	This Study
BHN418*pspC1LPxTG*	BHN418 *pspC1LpxTG::erm* Ery^R	This Study
BHN418Δ*lytB*	BHN418 *lytB::kan* Kan^R	This Study
BHN418Δ*pspC1*Δ*capsule*	BHN418 *pspC1::erm* Ery^R *Cps::tet* Tet^R	This Study
BHN418Δ*pspC2*Δ*capsule*	BHN418 *pspC2::erm* Ery^R *Cps::tet* Tet^R	This Study
BHN418Δ*pspC1*Δ*pspC2*Δ*capsule*	BHN418 *pspC1::erm* Ery^R *pspC2::kan* Kan^R *Cps::tet* Tet^R	This Study

[a] S1-Supplementary Reference 1

Supplementary Table 2. ***In vitro*** **binding of PspC1 and PspC2 to human FH measured by Surface Plasmon resonance/BIACORE.**

**Protein**	**k_a (1/Ms)**	**k_d (1/s)**	**K_D (nM)**	**Chi^2**
PspC1 BHN418	$6.9\ E^{+5}$	$6.2\ E^{-5}$	0.090	0.126
PspC2 BHN418	$4.1\ E^{+6}$	$5.5\ E^{-5}$	0.013	0.455
PspC D39	$4.8\ E^{+5}$	$4.3\ E^{-5}$	0.086	0.754

Supplementary Table 3. Primers used in this study.

Name of Primer	Sequence (5'to 3')
PspC2F_NdeI	CTTCTTCATATGACAGAGAAAGAGGGAAGTACC
PspC2R_XhoI	CTTCTTCTCGAGCTACTTCTTCGGTTTATTTGT
418ErmPspC1F	AAATATAGAAGGAAATAATTTTCGTGTTCGTGTGACTTGC
418ErmPspC1R	ATTAACTAGTTATATTAGGTATGTTGCTGATTAAGACGAG
418ErmPspC2F	AAATTATGGAGAATATAGACTTCGTGTTCGTGTGACTTGC
418ErmPspC2R	ATTTTTGAATGAATCAATCAATGTTGCTGATTAAGACGAG
418PspC1EF	TGCTCGTCTTAATCAGCAACATACCTAATATAACTAGTTAATAC
418PspC1ER	GTGCAAGTCACACGAACACGAAAATTATTTCCTTCTATATTTTT
418PspC2EF	TGCTCGTCTTAATCAGCAACATTGATTGATTCATTCAAAAATGT
418PspC2ER	GTGCAAGTCACACGAACACGAAGTCTATATTCTCCATAATTTTA
418PspC1KanF	AAATATAGAAGGAAATAATTTTCTACGGGGTCTGACGCTC
418PspC1KanR	ATTAACTAGTTATATTAGGTTTCTTAGAAAAACTCATCGAGCATC
418PspC1KF	GATGCTCGATGAGTTTTTCTAAGAAACCTAATATAACTAGTTAATAC
418PspC1KR	GAGCGTCAGACCCCGTAGAAAATTATTTCCTTCTATATTTTT
418PspC2F	GCTTGATAGCCTTGTATTCATGAG
418PspC2R	TCGATGAGGGCGAAAACCCTGTCT
418PspC1F	ATATAGGGTTAACATAAGTGTGTT
418PspC1R	TGGATCGAAAGTCTCATGAATACA
upcapsuleF	AATCCTTTGTTTTTTATAACCAAGGTT
upcapsuleR	TTCATGTGATTTTCCTCCATGATTAACACCTATACATTGAAC
DncapsuleF	TGTTCAATAAAATAACTTAGGTAGTAGAATGATTTTCTAGTC
DncapsuleR	TCCGTCCCATAACCAATGATTTAATGG
tetraF	GTTCAATGTATAGGTGTTAATCATGGAGGAAAATCACATGAA
tetraR	GACTAGAAAATCATTCTACTACCTAAGTTATTTTATTGAACAT
Asp2F	AAATATAGAAGGAAATAATTATGTTTAAATCAAATCATGAGCGAAGA
BC1R	GAGCGTCAGACCCCGTAGAAGTTTACCCATTCACCATTGGC
CCSP2	CTTCGCTCATGATTTGATTTAAACATAATTATTTCCTTCTATATTTTTTCT
DkanF	CCAATGGTGAATGGGTAAACTTCTACGGGGTCTGACGCTC
S2R	GGGGGTATTCCTACCACCAACCTCTTCCGCATGTACAACACTTCCCAT
S2C1	ATGGGAAGTGTTGTACATGCGGAAGAGGTTGGTGGTAGGAATACCC
Lpxtg1R	CTTGCTATTATCTGGTTTTTCTGCTTTTGG
Lpxtg1F	CCGGAAAAACCAAAACCAGCTGATCAACAA
Lpxtg2R	TTGTTGATCAGCTGGTTTTGGTTTTTCCGG
Lpxtg2F	CCAAAAGCAGAAAAACCAGATAATAGCAAG
D39PspCF_NdeI	CTTCTTCATATGACAGAGAACGAGGGAAGTACC
D39PspCR_BamHI	CTTCTTGGATCCCTATGCCATTGAACCATCAGTATTGTA
Pspc1BHN418F_NdeI	CTTCTTCATATGGAAGAGGTTGGTGGTAGGAATACCCCC
Pspc1BHN418R_XhoI	CTTCTTCTCGAGCTACGCCATTGAACCATCAGTATTGTA

Supplementary References

S1. Browall, S., Norman, M., Tangrot, J., Galanis, I., Sjostrom, K., Dagerhamn, J., Hellberg, C., Pathak, A., Spadafina, T., Sandgren, A., et al. Intraclonal variations among Streptococcus pneumoniae isolates influence the likelihood of invasive disease in children. J Infect Dis 209, 377-388 (2014).

S2. Hammerschmidt, S., Talay, S.R., Brandtzaeg, P., and Chatwal, G.S. SpsA, a novel pneumococcal surface protein with specific binding to secretory immunoglobulin A and secretory component. Mol Microbiol 25, 1113-1124 (1997).

S3. Janulczyk, R., Iannelli, F., Sjoholm, A.G., Pozzi, G., and Bjorck, L. Hic, a novel surface protein of Streptococcus pneumoniae that interferes with complement function. J Biol Chem 275, 37257-37263 (2000).

S4. Pei, J., Tang, M., and Grishin, N.V. PROMALS3D web server for accurate multiple protein sequence and structure alignments. Nucleic Acids Res 36, W30-34 (2008).
