# Supplementary material for: Factor H binding proteins protect division septa on encapsulated Streptococcus pneumoniae against complement C3b deposition and amplification
Source: Nat Commun. 2018 Aug 23;9:3398. doi: 10.1038/s41467-018-05494-w (PMC6107515; doi:10.1038/s41467-018-05494-w)
Supplement: Supplementary file 3 — Description of Additional Supplementary Files [file 41467_2018_5494_MOESM3_ESM.pdf]

Description of Additional Supplementary Files

File Name: Supplementary Data 1
Description: It shows full protein sequences of PspC1 and PspC2 in strain BHN418.
