# Supplementary material for: Factor H binding proteins protect division septa on encapsulated Streptococcus pneumoniae against complement C3b deposition and amplification
Source: Nat Commun. 2018 Aug 23;9:3398. doi: 10.1038/s41467-018-05494-w (PMC6107515; doi:10.1038/s41467-018-05494-w)
Supplement: Supplementary file 4 — Supplementary Data 1 [file 41467_2018_5494_MOESM4_ESM.pdf]

Supplementary Data 1. Full protein sequences of PspC1 and PspC2 in strain BHN418.

BHN418 PspC1:
MFASKSERKVHYSIRKFSIGVASVVVASLFLGGVVHAEEVGGRNTPTVTSSGQDISKKYADEVESHLKKILSEIQTQLDRKRHTKTV
ALINELQDIKKTYLYNLNVLKEKSELPSKIKAKLEVAFDQFKKDTLKPGEKVAEAEKKVAEAKKKAEDQKEEDRRNYPTNTYKTLEL
EIAESDVKVKEAELELVNEEAKPGNEEKIKKAKAKVESEKAEAIRLEEIKTDREEAKRKADAKLKEAVENNAATSEQGEPKRRVKRG
VLGEPATPDKKENDAKSSDSSVGEETLPSPSLKPEKKVAEAEKKAKDQKEEDRRNYPTNTYKTLELEIAESDVKVKEAELELVKEEA
KESRNEEKVKQAKAKVESKKAEATRLEKIKTDRKKAEEAKRKAAEEDKVKEKPAEQPQPAPAPQPEKPAPKPEKPAPAPKPENPA
EQPKAEKPADQQAEEDYARRSEEEYNRLTQQQPPKTEKPAQPSTPKTGWKQENGMWYFYNTDGSMATGWLQNNGSWYYL
NSNGAMATGWLQNNGSWYYLNANGSMATGWLQNNGSWYYLNANGSMATGWLQNNGSWYYLNANGSMATGWLQNN
GSWYYLNANGSMATGWLQYNGSWYYLNANGSMATGWLQYNGSWYYLNSNGAMVTGWLQNNGSWYYLNANGSMATD
WVKDGDTWYYLEASGAMKASQWFKVSDKWYYVNGSGALAVNTTVDSYRVNANGEWVN

BHN418 PspC2:
MFKSNHERRMRYSIRKFSVGVASVAVASLFMGSVVHATEKEGSTQAATSFNRGNGSQAEQRGELDLERDKAMKAVSEYVGK
MVRDAYVKSDRKRHKNTVALVNQLGNIKNRYLNEIVHSTSKSQLQELMMKSQSEVDEAVSKFEKDSFSSSSSGSSTKPETPQPE
NPEHQKPTTPSPDTKPSPQPEGKKPSVPDINQEKEKAKLAVVTYMSKILDDIQKHHLQKEKHRQIVALIKELDELKKQALSEIDNV
NTKVEIENTVHKIFADMDAVVTKFKKGLTQDTPKEPGNKKPSAPKPGMQPSPQPEVKPQLEKPKPEVKPQPEKPKPEVKPQPE
KPKPEVKPQPEKPKPEVKPQPEKPKPEVKPQPEKPKPEVKPQPEKPKPEVKPQPEKPKPEVKPQPEKPKPEVKPQPEKPKPEVKP
QPEKPKPEVKPQPEKPKPEVKPQPEKPKPEVKPQPEKPKPEVKPQPEKPKPEVKPQPEKPKPEVKPQPEKPKPEVKPQPEKPKPE
VKPQPEKPKPEVKPQPEKPKPDNSKPQADDKKPSTTNNLSKDKQPSNQASTNEKATNKPKKSLPSTGSISNLALEIAGLLTLAGA
TILAKKRMK
